# Analysis of muscle, hip, and subcutaneous fat in osteoporosis patients with varying degrees of fracture risk using 3T Chemical Shift Encoded MRI

**DOI:** 10.1016/j.bonr.2020.100259

**Published:** 2020-03-24

**Authors:** Dimitri Martel, Stephen Honig, Anmol Monga, Gregory Chang

**Affiliations:** a New York Langone Health, Department of Radiology, NYU School of Medicine, New York, USA; b New York Langone Health, Osteoporosis Center, Hospital for Joint Diseases, New York, USA

## Abstract

Osteoporosis (OP) is a major disease that affects 200 million people worldwide. Fatty acid metabolism plays an important role in bone health and plays an important role in bone quality and remodeling. Increased bone marrow fat quantity has been shown to be associated with a decrease in bone mineral density (BMD), which is used to predict fracture risk. Chemical-Shift Encoded magnetic resonance imaging (CSE-MRI) allows noninvasive and quantitative assessment of adipose tissues (AT). The aim of our study was to assess hip or proximal femoral bone marrow adipose tissue (BMAT), thigh muscle (MUS), and subcutaneous adipose tissue (SAT) in 128 OP subjects matched for age, BMD, weight and height with different degrees of fracture risk assessed through the FRAX score (low, moderate and high). Our results showed an increase in BMAT and in MUS in high compared to low fracture risk patients. We also assessed the relationship between fracture risk as assessed by FRAX and AT quantities. Overall, the results of this study suggest that assessment of adipose tissue via 3T CSE-MRI provides insight into the pathophysiology fracture risk by showing differences in the bone marrow and muscle fat content in subjects with similarly osteoporotic BMD as assessed by DXA, but with varying degrees of fracture risk as assessed by FRAX.

## 1 Introduction

Osteoporosis (OP) is a disease of fragile bones which increases fracture risk (Fx) in the aging population and represents an important public health problem. Fx risk assessment is commonly made using dual-energy x-ray absorptiometry (DXA), which allows assessment of the mineralized component of bone (both trabecular and cortical) via estimation of areal bone mineral density (BMD). This measurement allows the computation of a T-Score, which has been used as a means to define OP status (Hillier et al., 2011; Kim et al., 2019a; Elde and Madsen, 2019; Asirvatham et al., 2018; Choi et al., 2018).

However, DXA, as a 2-D planar technique, does not assess all the changes in bone tissue that can occur in the aging process and does not completely capture fracture risk. The T-score threshold used to define fracture risk and initiation of treatment is not optimal when applied to patients who are premenopausal, obese, diabetic, suffering from cancer, or corticosteroid therapy (Elde and Madsen, 2019; Leslie et al., 2018a; Leslie et al., 2018b; Majumdar et al., 2016; Leslie and Lix, 2014; Chen et al., 2012; Leslie et al., 2012; Lewiecki et al., 2011; Kanis et al., 2011). Indeed, this was the motivation for the development of FRAX, a risk calculator incorporating clinical factors (age, height, weight, etc.) that can be used to more accurately compute an individual's 10-year fracture risk and determine whether therapy should be initiated. Because DXA incompletely captures fracture risk, researchers have sought to identify other risk factors or therapeutic targets for OP.

An important component of bone tissue is bone marrow, which evolves throughout life and is the site of stem cells and marrow adipose tissue (BMAT) (Cawthorn et al., 2015; Scheller and Rosen, 2014; Cawthorn et al., 2014). An increase in BMAT has been associated with higher fracture risk in post-menopausal women and in men with osteoporotic BMD using Magnetic Resonance Spectroscopy (MRS) (Machann et al., 2017; Ruschke et al., 2017; Cordes et al., 2016; Di Pietro et al., 2016; Tufts et al., 2016; Ojanen et al., 2014; Karampinos et al., 2014; Patsch et al., 2013; Wang et al., 2012; Baum et al., 2012; Bredella et al., 2011; Li et al., 2011; Machann et al., 2008; Yeung et al., 2005; LeBlanc et al., 1999) or Chemical-Shift Encoded MRI (CSE-MRI) (Bray et al., 2018; Martel et al., 2018; Nemeth et al., 2018a; Martel et al., 2019; Bray et al., 2018; Wang et al., 2016; Bolan et al., 2013; Maas et al., 2001). MRS is a mono-voxel method that allows quantitative measurement of the fat spectrum and assessment not only of fat quantity in the voxel but also fat composition. However, this method is time-consuming, not widely used in the clinic, only permits quantification in pre-specified small regions-of-interest, and requires advanced postprocessing. On the contrary, CSE-MRI, which is a Dixon-based method (Maas et al., 2001; van Vucht et al., 2019; Dixon, 1984) permits separation of fat and water in images based on echo-time phase variation between fat and water spins. Using two or three echoes, fat-water separation is feasible using a post-processing method such as IDEAL and has been used mainly for quantification of hepatic adipose tissue (Corrias et al., 2018; Min et al., 2018; Ajmera et al., 2018; Wildman-Tobriner et al., 2018; Donato et al., 2017), subcutaneous adipose tissue (Nemeth et al., 2018a; Abildgaard et al., 2018; Hamilton et al., 2017; Hamilton et al., 2017; Hu et al., 2016), muscle (Duijnisveld et al., 2017; Burakiewicz et al., 2017; Yoo et al., 2015), and more recently to study bone (Martel et al., 2018; Martel et al., 2019; van Vucht et al., 2019; Singhal and Bredella, 2019; Karampinos et al., 2018; Gregory et al., 2017). Moreover, CSE-MRI is widely accepted and commonly offered as a standard fat-suppression technique and available on nearly every MRI scanner from all vendors.

Aging is accompanied by a loss of subcutaneous fat and accumulation of lipids in bone marrow and skeletal muscle (Kirkland et al., 2002; Caso et al., 2013). This accumulation has been studied mainly in vertebrae and associated with lower BMD and increased vertebral fracture (Schwartz et al., 2013) (Blake et al., 2009) as a predictor for osteoporosis and osteopenia (Kim et al., 2019b). In addition, changes in trunk muscle has been studied and shown that increasing fat deposition in muscle is driven primarily by age, rather than BMI in women. More recently, studies suggest a link between BMAT and energy metabolism (Lecka-Czernik, 2012; Suchacki and Cawthorn, 2018; Cornish et al., 2018; Li et al., 2019). These findings show a possible link between different adipose tissues and bone quality which can be assessed through a non-invasive, accurate, and quantitative method such as CSE-MRI.

The purpose of our study was to use CSE-MRI measurements to quantify the adipose tissue content in the hip, muscle, and subcutaneous tissue and determine the correlation between regional adipose tissue content with level of fracture risk in OP subjects as assessed by FRAX.

## 2 Material/methods

### 2.1 Subjects and FRAX score

This prospective, HIPAA compliant study was approved by the institutional review board, and we obtained written informed consent from all subjects. One hundred twenty-eight female subjects were recruited (mean age = 61.12 ± 7.22 years, range = 42–79 years; mean body mass index = 22.08 ± 3.57) from our institution with total hip dual-energy X-ray absorptiometry (DXA, GE Lunar, Rahmay, NJ) results consistent with osteoporosis (mean total hip BMD T-score = −2.872 ± 0.552) during a two years period. FRAX scores were computed according to the standard method (https://www.sheffield.ac.uk/FRAX/tool) considering patient race. Patients were then divided into three groups for analysis based upon overall FRAX score: low (LOW, FRAX score for major osteoporotic fracture <10), moderate (MOD, FRAX score for major osteoporotic fracture >10 and < 20) and high (HIGH, FRAX score for major osteoporotic fracture >20).

### 2.2 Magnetic resonance imaging protocol

All MRI scanning was performed on a 3T MRI scanner (SKYRA system, Siemens Healthcare) using an 18-channel flexible coil overlying the pelvis. A 3D sagittal spoiled gradient echo sequence with a monopolar flyback readout gradient was used to acquire data at multiple echo-times. The acquisition parameters were: TR/FA 16 ms/3°; 3 or 6 echo times (2.1/2.8/3.5 during year one of data acquisition and 2.1/2.8/3.5/4.5/5.2/5.9 ms during the subsequent years of data acquisition) were acquired; Bandwidth = 1400 Hz.pixel^−1^; FOV = 330 mm^2^ in-plane to cover both hips from the level of the femoral head to the femoral shaft; matrix = 128 × 128; 40 slices; slice thickness = 5 mm, total scan time = 6 min. Raw data were systematically saved.

### 2.3 IDEAL reconstruction

Images were reconstructed from raw data with coil sensitivity correction (Walsh et al., 2000) using Matlab routines (MATLAB2019b, The Mathworks, The MathWorks Inc., Natick, MA, USA). The IDEAL algorithm (Hu et al., 2012; Reeder et al., 2005) was then used for fat/water separation using an eight peaks fat spectrum model and T2* estimation. Fat (F), and Water (W) parametric maps were then obtained, and Proton Density Fat Fraction (PDFF) was then computed according to the relation PDFF = F/(F + W).

### 2.4 Segmentation of AT

**Fig. 1:**
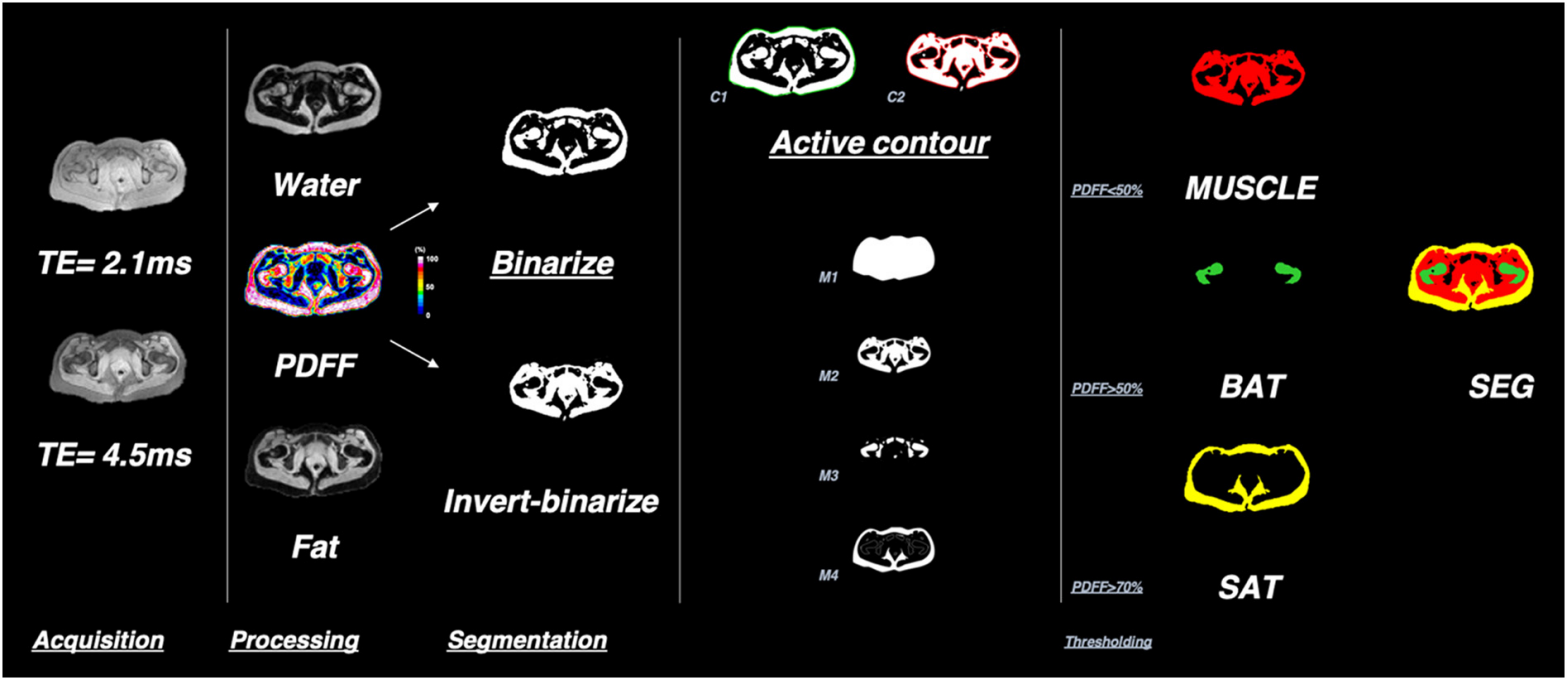
Image reconstruction workflow. Acquired raw data are processed to reconstruct images for each of the acquired echoes (example for echoes acquired at 2.1 and 4.5 ms). IDEAL is then used to separate fat and water in the image. From these, PDFF maps are computed, and segmentation of each AT depot is performed.

Segmentation was performed using in-house Matlab 2019b routines on 20 slices chosen to be centered on femoral head. Briefly, a mask based on threshold of Fat and Water maps was obtained to suppress image background and applied to PDFF map. This latter was then binarized to separate high fat quantity tissue (SAT and BMAT) from low fat quantity tissue (muscle) and an active contour was then applied (68) on binarized (C1) and inversed binarized (C2) PDFF maps. From these contours, masks were defined: M1 from C1, M2 from C2, M3 from inversed M2 and M4 from difference between M1 and M2. Adipose tissue depots were then segmented by thresholding PDFF maps in the obtained mask (<50% for muscle (MUS) in M2 and >50% for BMAT in M3 and >70% subcutaneous adipose tissue (SAT) in M4). AT masks were inspected to assure a morphological connectivity of pixel binary masks for each adipose tissue (Fig. 1).

### 2.5 Statistics

Statistical analysis was performed using the MATLAB 2019b Statistics and Machine Learning Toolbox and GraphPad Prism 8 software for data representation.

#### 2.5.1 Demographics

A Kruskal-Wallis parametric one-way analysis of variance was performed on demographic data to test the difference in the distribution for each group with a threshold of p < 0.05.

AT depots: Masks were used to integrate PDFF maps and volume of each tissue (VOL) was assessed as the ratio of number of voxels in each tissue mask and total number of voxels in associated image.

The null hypothesis was set as the mean of the distribution of each dataset is the same. A Brown-Forsythe unpaired one-way analysis of variance (ANOVA) test with Welch's correction was used to assess significant differences between groups in each AT depot with a threshold of p < 0.05 to reject the null hypothesis.

#### 2.5.2 Regression analysis

Demographic data, PDFF R2*, and volume for each AT depots were normalized and used as predictor variables to create a linear regression model with interaction by stepwise regression with FRAX score as the response variable (Eq. 1).

(1) $$Y=\alpha +\beta_{1}x_{1}+\beta_{2}x_{2}+\beta_{3}x_{3}\ldots +\gamma_{1}\left(x_{1}\times x_{2}\right)+\gamma_{2}\left(x_{1}\times x_{3}\right)+\gamma_{3}\left(x_{2}\times x_{3}\right)+$$

With Y the response variable, x the predictors, α the slope, β the linear intercept coefficients and γ the intercept coefficient of two-way interaction terms.

The stepwise regression is a bidirectional model selection approach in which predictor variables are considered initial predictors of FRAX. A new model is then obtained by iteratively adding or subtracting variables and/or their association (interaction variables) based on their predictive power according to the Akaike information criterion (AIC) (Akaike, 1974). To prevent overfitting k-fold cross-validation method was used: data were divided into k = 5 folds for cross-validation (Model was trained on 80% and tested on 20% of the dataset). The ANOVA test and Bland-Altmann methodology were used to examine the quality of the fitted model.

#### 2.5.3 Covariance

Covariances between PDFF and demographic data were computed using Pearson r-square values within each group. p-Values were calculated, assuming the null hypothesis that the data were sampled from a population where there is no correlation between the two variables with a threshold of p < 0.05 to reject the null hypothesis.

## 3 Results

The demographic data of subjects included in this study are shown in Table 1 including age, height, weight, BMI. Subjects were divided into three groups depending on their FRAX score. No differences between the groups were found in terms of age, height, weight, and BMI.

**Table 1:** Demographics of subjects, *: p < 0.05.

	Low (n = 42)					Moderate (n = 52)					High (n = 34)				
	Height (m)	Weight (kg)	BMI (kg/m^2^)	Age (years)	FRAX	Height (m)	Weight (kg)	BMI (kg/m^2^)	Age (years)	FRAX	Height (m)	Weight (kg)	BMI (kg/m^2^)	Age (years)	FRAX
Minimum	1.5	42	17	41	2.8	1.4	38	16	49	11	1.5	42	17	54	20
Maximum	1.8	95	38	72	10	1.8	85	30	79	19	1.8	96	30	78	50
Range	0.35	52	20	31	7.2	0.40	48	14	30	8.0	0.29	54	13	24	30
95% CI of median															
Actual confidence level	96%	96%	96%	96%	96%	96%	96%	96%	96%	96%	98%	98%	98%	98%	98%
Lower confidence limit	1.6	52	21	54	7.2	1.6	49	20	59	12	1.6	54	21	62	22
Upper confidence limit	1.7	61	23	58	8.5	1.6	58	22	65	16	1.7	61	24	66	26
Mean	1.6	59	23	57	7.6*	1.6	56	22	62	14*	1.6	58	22	64	25
Std. deviation	0.080	12	4.1	6.9	1.9	0.082	12	3.4	6.9	2.7	0.067	9.7	3.1	5.8	6.3
	0.012	1.8	0.63	1.1	0.29	0.011	1.6	0.47	0.96	0.37	0.011	1.7	0.53	1.0	1.1
Coefficient of variation	4.9%	20%	18%	12%	25%	5.1%	21%	16%	11%	19%	4.1%	17%	14%	9.1%	25%

Fig. 1 shows the image reconstruction workflow used for processing of the data, including image reconstruction, fat-water separation, PDFF map generation, and the segmentation of AT depots into BMAT, MUSCLE, and SAT using the active contour method and thresholding of PDFF maps.

**Fig. 2:**
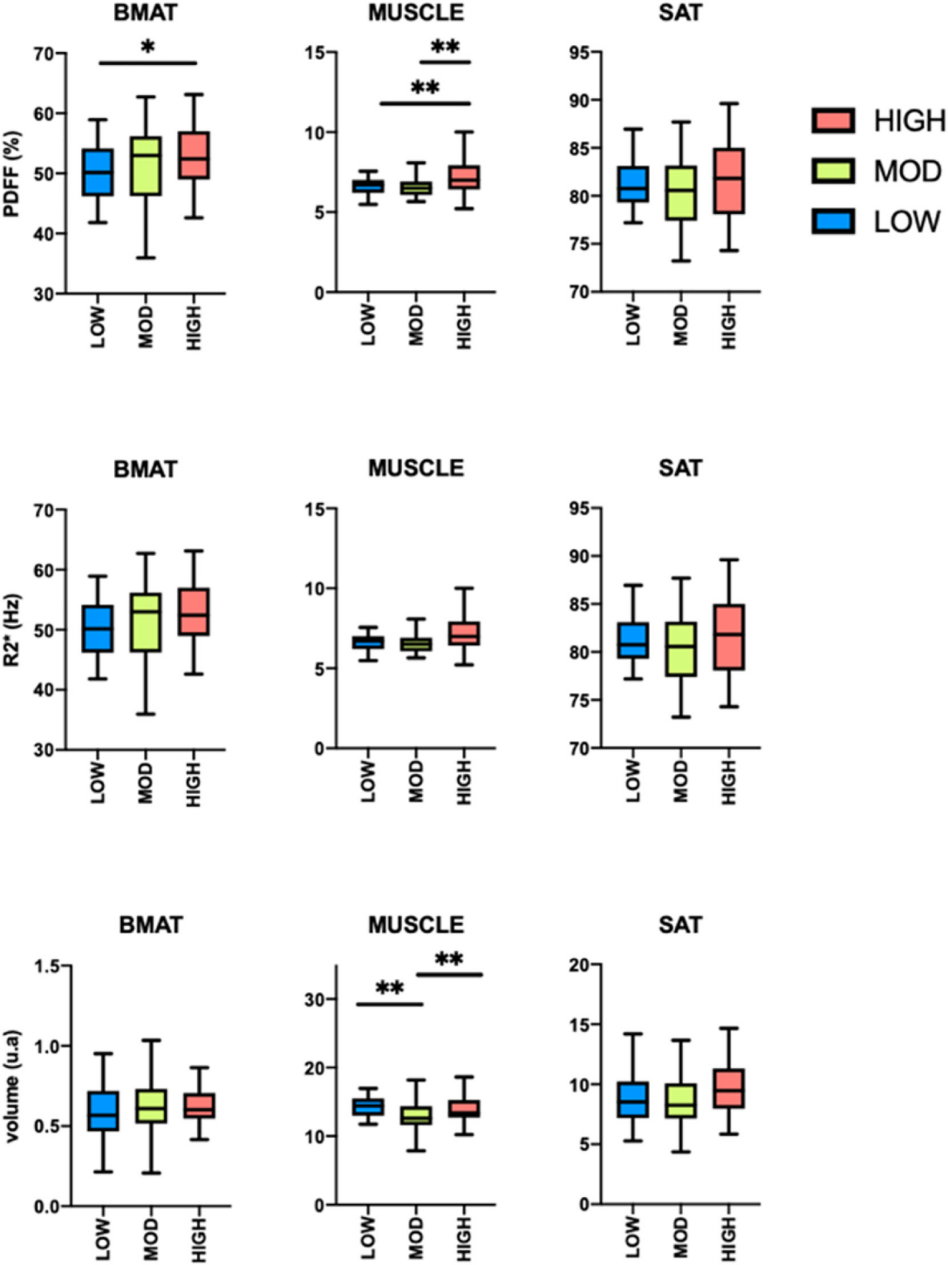
Box and whisker plots of PDFF, R2* and volume in proximal femoral bone marrow adipose tissue (BMAT), thigh muscle (MUSCLE), and subcutaneous adipose tissue (SAT). (On each box, the central mark indicates the median, and the bottom and top edges of the box indicate the 25th and 75th percentiles, respectively. Statistical significance threshold *: p < 0.05, **: p < 0.01).

Fig. 2 presents the results of fat quantification within each AT depot. In BMAT, we found a higher quantity of PDFF in HIGH subjects compared to LOW subjects (+5%, p = 0.032). In muscle tissue, we found a higher quantity of PDFF in HIGH compared to both LOW (+8.87%, p = 0.008) and MOD subjects (+9.25%, p = 0.006). There were no differences between groups with regards to R2* measured in any of the AT depots.

**Fig. 3:**
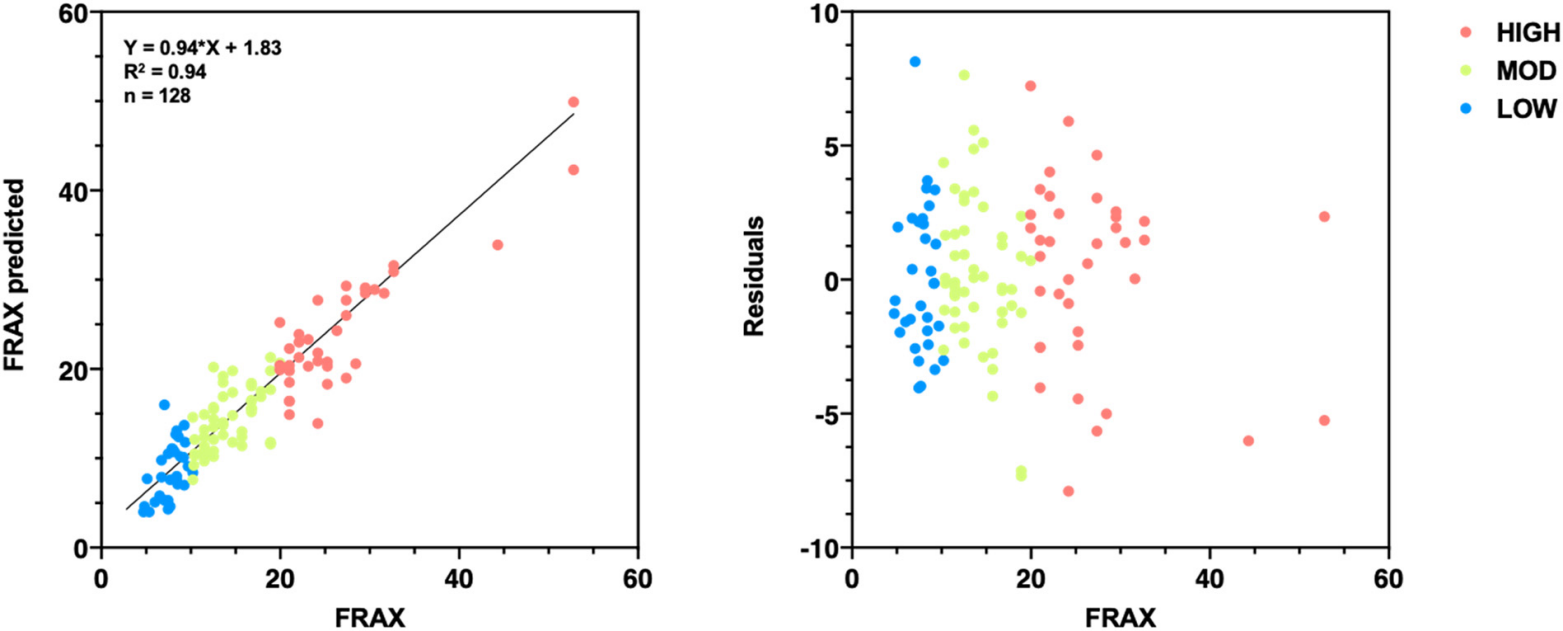
a) Plot of predicted FRAX versus actual FRAX score and b) residual of multiple linear regression performed using a two-way interaction model, including MRI parameters and demographic data.

Table 2 presents regression analysis performed using an interaction model with demographic data and MRI parameters. The results of the regression indicated the two predictors explained 88.4% of the variance (R^2^ = 0.943, R^2^_adjusted_ = 0.884, F(1,12) = 13.4, p < 0.001). The model is significant at the 5% significance level (Fig. 3).

**Table 2:** Predictor variables in multiple linear regression model (according to Wilkinson and Rogers's notation for symbolic description of factorial models). Terms used in the model are highlighted in bold.

Predictor variable	Estimate	SE	p-Value	95% CI	
**PDFF_BAT**	1.269	0.189	***0.001***	0.890	1.648
**PDFF_MUS**	1.219	0.501	**0.019**	0.214	2.224
**PDFF_SAT**	−0.707	0.350	**0.049**	−1.410	−0.004
**T2_BAT**	−78.975	16.378	***0.001***	−111.871	−46.078
T2_MUS	4.452	2.468	0.077	−0.506	9.410
**T2_SAT**	−24.508	8.574	***0.006***	−41.729	−7.286
VOL_BAT	0.138	0.234	0.558	−0.332	0.607
VOL_MUS	−0.312	0.195	0.117	−0.704	0.081
VOL_SAT	−0.585	0.581	0.318	−1.752	0.581
HEIGHT	0.326	0.249	0.197	−0.175	0.827
**WEIGHT**	0.762	0.302	**0.015**	0.155	1.369
**AGE**	−0.837	0.189	***0.001***	−1.215	−0.458
**PDFF_BAT:T2_BAT**	43.529	12.016	***0.001***	19.394	67.664
**PDFF_BAT:T2_MUS**	−14.219	2.377	***0.001***	−18.994	−9.444
**PDFF_BAT:HEIGHT**	−0.912	0.287	***0.003***	−1.488	−0.336
**PDFF_BAT:WEIGHT**	−1.166	0.388	***0.004***	−1.945	−0.386
**PDFF_MUS:PDFF_SAT**	0.977	0.425	**0.026**	0.124	1.829
**PDFF_MUS:T2_BAT**	167.200	27.013	***0.001***	112.946	221.461
**PDFF_MUS:T2_MUS**	−24.042	3.758	***0.001***	−31.590	−16.495
PDFF_MUS:VOL_BAT	−1.362	0.705	0.059	−2.778	0.054
**PDFF_MUS:VOL_MUS**	−1.226	0.435	***0.007***	−2.100	−0.352
**PDFF_MUS:VOL_SAT**	−2.093	0.670	***0.003***	−3.439	−0.746
**PDFF_MUS:HEIGHT**	1.148	0.416	***0.008***	0.312	1.984
**PDFF_MUS:WEIGHT**	−1.544	0.560	***0.008***	−2.669	−0.418
**PDFF_SAT:T2_MUS**	11.093	2.745	***0.001***	5.580	16.606
**PDFF_SAT:T2_SAT**	−18.890	6.482	***0.005***		−5.871
**PDFF_SAT:VOL_BAT**	−1.362	0.511	**0.010**	−2.389	−0.335
**PDFF_SAT:VOL_MUS**	−1.357	0.313	***0.001***	−1.986	−0.728
**PDFF_SAT:HEIGHT**	0.860	0.373	**0.025**	0.111	1.609
**PDFF_SAT:AGE**	0.987	0.304	***0.002***	0.375	1.598
**T2_BAT:T2_MUS**	−167.500	45.552	***0.001***	−258.997	−76.008
**T2_BAT:T2_SAT**	−677.950	92.430	***0.001***	−863.603	−492.299
**T2_BAT:VOL_BAT**	−108.170	28.580	***0.001***	−165.578	−50.771
**T2_BAT:VOL_MUS**	66.749	17.767	***0.001***	31.063	102.436
**T2_BAT:WEIGHT**	38.120	14.300	**0.010**	9.398	66.843
**T2_MUS:T2_SAT**	180.210	29.053	***0.001***	121.852	238.560
**T2_MUS:VOL_BAT**	19.327	3.468	***0.001***	12.361	26.292
**T2_MUS:VOL_MUS**	−6.458	2.670	**0.019**	−11.822	−1.094
**T2_MUS:VOL_SAT**	6.224	2.866	**0.035**	0.468	11.980
**T2_MUS:HEIGHT**	12.309	2.166	***0.001***	7.959	16.660
**T2_MUS:WEIGHT**	−11.416	2.830	***0.001***	−17.100	−5.733
**T2_SAT:VOL_BAT**	98.360	13.594	***0.001***	71.055	125.665
**T2_SAT:VOL_MUS**	26.011	10.814	**0.020**	4.292	47.731
T2_SAT:HEIGHT	−12.116	6.938	0.087	−26.051	1.819
**VOL_BAT:VOL_SAT**	−1.691	0.506	***0.002***	−2.708	−0.674
**VOL_MUS:VOL_SAT**	1.963	0.480	***0.001***	1.000	2.927
**VOL_MUS:HEIGHT**	−0.654	0.220	***0.005***	−1.097	−0.212
**VOL_MUS:WEIGHT**	0.860	0.264	***0.002***	0.330	1.389
**VOL_MUS:AGE**	1.093	0.239	***0.001***	0.612	1.574
**VOL_SAT:HEIGHT**	−1.258	0.434	***0.006***	−2.129	−0.387
**VOL_SAT:WEIGHT**	3.494	0.534	***0.001***	2.422	4.567
VOL_SAT:AGE	0.750	0.419	0.079	−0.091	1.592
**HEIGHT:WEIGHT**	−0.688	0.185	***0.001***	−1.060	−0.317

Statistical significance is highlighted for p<0.05 (bold) and p<0.01 (italic).

**Fig. 4:**
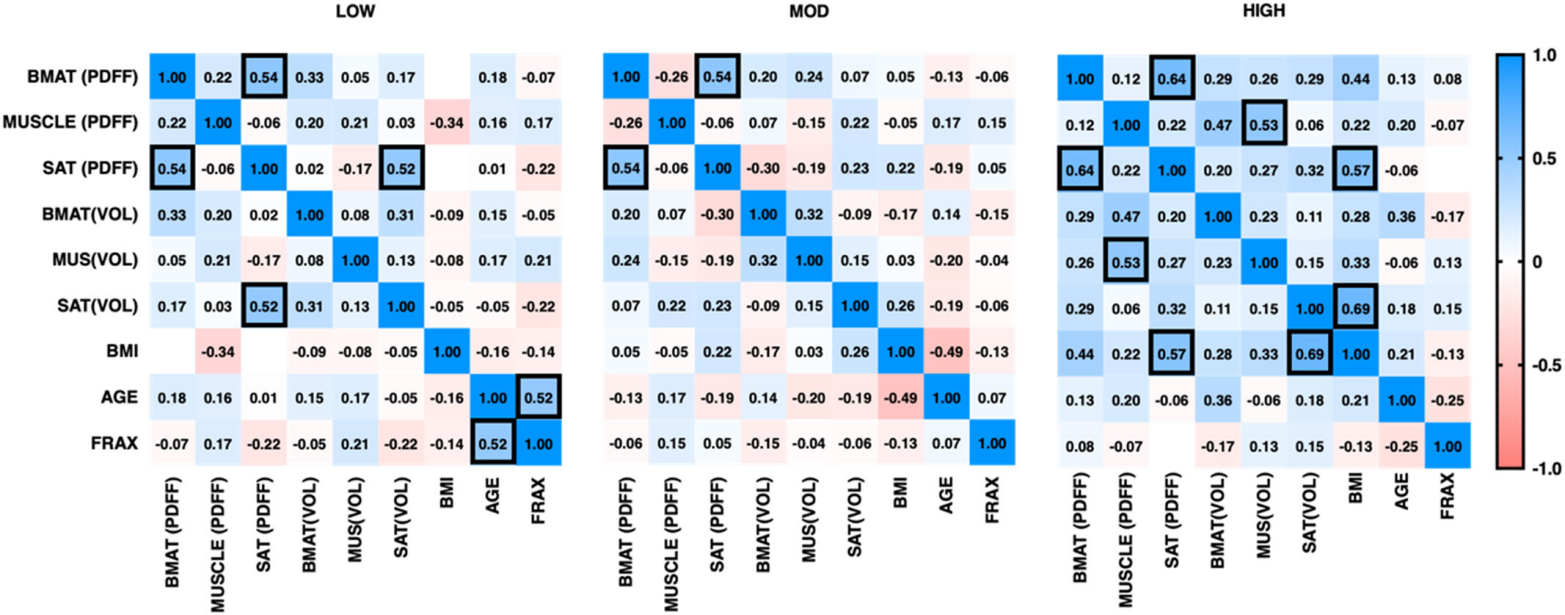
Correlation matrix between MRI parameters measured for each group (significant correlations highlighted, p < 0.05).

Fig. 4 presents the Pearson correlation coefficients computed between parameters for each group. We listed significant correlation (r < −0.5 or > 0.5 and p < 0.05) between MRI parameters within each of the fracture risk groups. Within all groups, BAT PDFF and SAT PDFF appear to have a moderate correlation (r = 0.54, p < 0.01 for LOW, r = 0.54, p < 0.01 for MOD and r = 0.64 for HIGH).

Within the LOW group, there was a moderate correlation between SAT PDFF and SAT VOL (r = 0.52, p < 0.01), FRAX and age ((r = 0.52 (p < 0.01)). In the HIGH group we found moderate correlation between MUS PDFF and MUS VOL (r = 0.53 (p < 0.01)), high correlation between SAT PDFF and BMI (r = 0.57 (p < 0.01)), SAT VOL and BMI (r = 0.69 (p < 0.01)).

## 4 Discussion

In summary, we have shown that 3T CSE-MRI permits quantification of AT depots in OP subjects and that the AT tissue in proximal femur marrow and thigh muscle differ depending on a patients' fracture risk. To the best of our knowledge, this study was the first to investigate the correlation between AT depots using CSE-MRI and osteoporotic fracture risk. Overall, the CSE-MRI method can permit a better understanding of the OP disease process, notably in terms of the cellular composition of bone marrow and, thus, its metabolic activity (Fukuda et al., 2019). The method can be extended to other musculoskeletal diseases such as sarcopenia (Reiss et al., 2019; Beaudart et al., 2019; Locquet et al., 2018; Santos et al., 2018) and also other diseases of increased fracture risk such as systemic lupus erythematosus (Chalayer et al., 2017; Bultink and Lems, 2016; Edens and Robinson, 2015).

Overall, our results are novel because they help link metabolic information about bone and muscle AT content measured in the hip through MRI and major osteoporotic fracture risk assessed via FRAX in subjects who have osteoporotic bone mineral density in the hip. The results suggest that even among patients with similarly osteoporotic BMD, bone marrow fat content and muscle tissue fat content can vary depending on clinical fracture risk. Though the results should be validated in future studies, the results suggest that assessment of bone and muscle tissue fat content might be used as a way to further stratify patients' fracture risk, beyond simply measuring BMD via DXA.

Our results are congruent with prior studies, which have shown changes in bone marrow fat content in vertebrae in subjects with varying BMDs. Griffith et al. (Griffith et al., 2006) used MRS and found a significant increase in lumbar vertebral bone marrow fat content in women with osteoporosis compared to those with normal BMD. Specifically, vertebral marrow fat fraction was significantly increased in osteoporotic subjects (67.8 ± 8.5%) compared with healthy subjects (59.2 ± 10.0%). Similar results were found in another study of men: bone marrow fat fraction was significantly increased in subjects with osteoporosis (58.2 ± 7.8%) and osteopenia (55.7 ± 10.2%) compared to subjects with normal BMD (50.5 ± 8.7%) (Griffith et al., 2005). We have built upon Griffith's work by performing our study in the hip using CSE MRI and also comparing our results to fracture risk as assessed by FRAX, rather than comparing results to BMD only. We do note that Griffith's work was published before FRAX became widely available in the late 2000s.

Our study associates fracture prediction risk in osteoporosis patients using CSE-MRI parameters in muscle, bone marrow, and subcutaneous adipose tissue. Few prospective studies have investigated the association between AT assessed by DXA and fracture risk, and results have been inconsistent (Malkov et al., 2015; Zaslavsky et al., 2017; McLean et al., 2018; Codari et al., 2020). This latter can be explained by the use of DXA to assess AT, as recently pointed by Tavoian et al. (Tavoian et al., 2019), which found no association between the measurement of lean mass when compared to MRI as the gold-standard.

Our regression model shows a linear trend with the FRAX score. Specifically, we found that 88% of the variability of the FRAX score was accounted for by MRI parameters pertaining to fat. Importantly, the lack of a unitary R-squared value suggests that MRI parameters provide different information about patient health than the information provided by FRAX. This makes senses as DXA does not capture information about bone marrow or muscle adipose tissue content. The model used in this study is an interaction model with 12 predictors: PDFF, R2*, volume about AT depots, age, height, and weight. BMI was not included in the model because of its high correlation with height and weight. These predictor variables have a low correlation within each other for MRI parameters.

The correlation matrix computed for each group showed significant (p < 0.01) moderate correlations. BAT PDFF and SAT PDFF were correlated within each group, which suggests a parallel development of these ATs with the fracture risk. We also found correlations between SAT PDFF and SAT VOL, MUS PDFF, and MUS VOL, which may indicate an expansion of these ATs with increased fracture risk (Hamrick et al., 2016). We note that we only used multiple linear regression. More complex machine learning methods such as a support vector machine or Gaussian process regression models could be applied for fracture risk prediction using multiparametric MRI, DXA, FRAX scores and/or other clinical data. These more complex methods were not tested since they require a larger dataset than available in this study.

The advantage of the CSE method compared to MRS is that it provides large volume coverage, permitting assessment of any region of interest within this volume, and can be performed in less than 5 min of scan time, which makes it suitable for clinical scanning. The CSE method that we used has previously been validated with magnetic resonance spectroscopy in vivo, demonstrating a strong measurement agreement (Karampinos et al., 2018). We did assume that the fat spectrum is similar in muscle, BMAT, and SAT. Hamilton et al. (2017) have shown that there can be differences in terms of fat composition between deep and surface SAT using MRS notably. While the CSE method has been validated in the proximal femur with MRS, in future work, it will be important to perform the same validation in muscle and SAT. In the future, fat/water separation quality can be enhanced by the use of more recent sequences such as volume interpolated Dixon (van Vucht et al., 2019; Yoo et al., 2015; Morani et al., 2015; Yu et al., 2013; Vogt et al., 2005; Rofsky et al., 1999) or increasing the number of echoes (Franz et al., 2019).

The present study has limitations. First, the T2 relaxation of the multipeak fat spectrum can impact PDFF estimation, notably because each component of the spectrum has its own T2 (Bydder et al., 2008; Yang et al., 2014). The use of multiple-TE MRS in each AT depot could be used to acquire a T2* calibrated fat spectrum model in each AT depot. This would be time-consuming. In addition, in our study, the TEs used to acquire the data were much shorter than T2, which limits the impact of this confounder. Next, no differentiation between deep and surface subcutaneous adipose tissue was considered. Due to the high content in the fat of theses tissues, it can be difficult to evaluate a change. Lundbom et al. (Lundbom et al., 2016) found a distinct association between intramyocellular fatty acids in and deep subcutaneous AT in obese patients using MRS, and more recently, Hamilton et al. found differences between theses tissues in term of composition (Hamilton et al., 2017). We note that more advanced CSE-MRI methods may provide information about fat composition with the same precision as MRS (Martel et al., 2018; Nemeth et al., 2018a; Martel et al., 2019; Peterson and Mansson, 2013; Nemeth et al., 2018b; Leporq et al., 2013; Leporq et al., 2014; Leporq et al., 2017).

## 5 Conclusion

In conclusion, assessment of tissue fat via 3T CSE-MRI provides insight into the pathophysiology of fracture risk by showing differences in the bone marrow and muscle fat content in subjects with similarly osteoporotic BMD as assessed by DXA, but with varying degrees of fracture risk as assessed by FRAX. The 3T CSE-MRI method advantageously provides large volume coverage and can be performed in clinically feasible scan times (<6 min). 3T CSE-MRI could be used in the future to study the relationship between adipose tissue and bone health and possibly even provide an additional surrogate marker of fracture risk beyond DXA/FRAX if the results are validated in larger studies. 3T CSE-MRI could also be used in the future to assess whole-body AT and could be applied to better understand metabolic interactions between fat depots throughout the body (e.g. liver, muscle, bone marrow, subcutaneous tissue) in diseases in which there is alteration in tissue lipid content (e.g., sarcopenia, lupus, obesity, heart disease).

## Declaration of competing interest

D.M. has no conflict of interest

S.H. has no conflict of interest

A.M. has no conflict of interest

G.C. has consulted for Regeneron and Guidepoint for work unrelated to this study.

G.C. has also applied for a patent for technology unrelated to this study.
